# Current Imaging Techniques for Lymph Node Staging in Prostate Cancer: A Review

**DOI:** 10.3389/fsurg.2018.00074

**Published:** 2018-12-07

**Authors:** Raoul Muteganya, Serge Goldman, Fouad Aoun, Thierry Roumeguère, Simone Albisinni

**Affiliations:** ^1^Department of Nuclear Medicine, Erasme Hospital, Université Libre de Bruxelles, Brussels, Belgium; ^2^Urology Department, Institut Jules Bordet, Université Libre de Bruxelles, Brussels, Belgium; ^3^Urology Department, Hôtel Dieu de France, Université Saint Joseph, Beyrouth, Lebanon; ^4^Urology Department, Erasme Hospital, Université Libre de Bruxelles, Brussels, Belgium

**Keywords:** imaging, MRI, lymph nodes, PET, PSMA, prostate cancer

## Abstract

**Introduction:** Lymph node metastases (LNM) represent a proven prognostic factor for biochemical recurrence (BCR)-free survival, metastatic free survival and overall survival in prostate cancer (PCa). Although pelvic node dissection remains the gold standard for the detection of LNM, novel imaging techniques are entering clinical practice, in the effort to improve LNM detection and spare unnecessary surgeries. Aim of the current review is to describe such imaging techniques and explore their advantages and limitations.

**Evidence Acquisition:** The National Library of Medicine Database was searched for relevant articles published between January 2013 and August 2018. A wide search was performed including the combination of following words: “Prostate” and “Cancer” and “staging” and “Lymph Node” and “imaging” and (“MRI” or “PET”). The initial list of selected papers was enriched by individual suggestions of the authors of the present review.

**Evidence Synthesis:** DWI-MRI in detection of lymph node invasion has a sensitivity and specificity of 41 and 94%, respectively. For SPIO MRI using ferumoxtran-10, the sensitivity for detection of LNM with short axis diameter of 5–10 mm is reported at 96.4%, compared to 28.5% with MRI alone. PSMA PET/CT is growing exponentially, both in the initial detection of LNM and for BCR evaluation. Fluciclovine PET could improve detection of subcentimetric pathologic lymph nodes. Sentinel lymph node techniques remain experimental and not validated in the field of PCa.

**Conclusions:** Molecular imaging, particularly PSMA ligand PET imaging, present interesting diagnostic accuracy in LN diagnosis even in subcentimetric LN. DWI-MRI yields good results in LN involvement evaluation and the use of contrast agent such SPIO may improve the detection rate. The SLN technique is limited to experimental protocols and for intermediate or high-risk PCa. Prospective trials are awaited to evaluate the true clinical impact of these imaging techniques on PCa oncologic outcomes.

## Introduction

In men, prostate cancer (PCa) represents the second cause of cancer and the fifth leading cause of cancer-related death worldwide (1). Tumor lymph node metastasis (LNM) is proven to be a prognostic factor for biochemical recurrence (BCR)-free survival, metastatic free survival and overall survival (OS) in PCa (2). Currently, pelvic lymph node dissection (PLND) is the gold standard for the evaluation of the lymph node extension and is recommended for patients harboring high-risk prostate cancer and also for intermediate risk if the estimated risk for a positif lymph node invasion exceed 5% (3). Multiple validated nomograms (4, 5) are currently employed by surgeons to decide whether PLND should be performed. The accepted recommendation is that if risk of LNM exceeds 5%, a PLND should be performed at the time of radical prostatectomy (3). Nonetheless, PLND is not devoid of complications (6, 7) and to date many patients undergo the procedure although harboring specimen confined disease, with limited oncologic advantage and undoubted increase in surgical time and morbidity (8, 9). Various radiological and nuclear medicine imaging techniques have been proposed to evaluate the pre-operatory lymph node staging but also for the evaluation of tumor lymph node invasion in case of biochemical recurrence (BCR). However, to date, the ideal exam does not yet exist: indeed, a highly sensitive imaging exam, limiting false negative cases, would be greatly welcomed by the urologic surgical community, in order to identify those patients who truly need PLND, and sparing the others from the procedure.

The aim of this review is to give an overview of the role of radiological and molecular imaging currently used in lymph node staging for PCa, and to explore future directions of research in this field.

## Evidence Acquisition

The National Library of Medicine Database was searched for relevant articles published between January 2013 and August 2018. A wide search was performed including the combination of following words: “Prostate” AND “Cancer” AND “staging” AND “Lymph Node” AND “imaging” AND (“MRI” OR “PET”). Although recent articles were prioritized, works with relevant historical findings were referenced if necessary. Only publications in English language were considered and evidence was limited to human data. Each article's title, abstract and text were reviewed for their appropriateness and their relevance. The initial list of selected papers was enriched by individual suggestions of the authors of the present review. Table 1 presents a synoptic view of available techniques.

**Table 1:** Different imaging techniques for lymph node staging in prostate cancer.

**References**	**Technique**	**N**°** patients**	**Design**	**Risk of LNM**	**Gleason score**	**Sensibility (%)**	**Specificity (%)**	**PPV (%)**	**NPV (%)**
Hövels et al. (10)	CT scan	1,024 (18 studies with CT included)	Meta-analysis	All	n.m	42	82	n.m	n.m
Hövels et al. (10)	MRI alone	628 (10 studies with MRI included)	Meta-analysis	All	n.m	39	82	n.m	n.m
Von Below et al. (11)	MRI-DWI	40	Prospective study	Intermediate and high risk	≥6	41	94	70	82
Harisinghani et al. (12)	MRImp + SPIO	80	Prospective study	All	5–8	100	95.7	94.2	100
Beauregard et al. (13)	^18^F-FDG PET	44	Prospective study	High risk	≥8	27–47	n.m	n.m	n.m
Chang et al. (14)	^18^F-FDG PET	24	Prospective study	n.m	n.m	75	100	100	67.7
Evangelista et al. (15)	^11^C-/and ^18^F-Choline PET	441 (10 studies included)	Meta-analysis	All	n.m	58	94	n.m	n.m
Tilki et al. (16)	^18^F-FEC PET	56	Prospective study	n.m	≥6	39.7	95.8	75.7	83
Schumacher et al. (17)	^11^C-Acetate PET	19	Prospective study	n.m	≥7	90	67	75	86
Haseebuddin et al. (18)	^11^C-Acetate PET	107	Prospective study	Intermediate and high risk	≥6	68	78.1	48.6	88.9
Budäus et al. (19)	^68^Ga-PSMA PET	30	Retrospective study	High risk	≥7	33.3	100	100	69.2
Zang et al. (20)	^68^Ga-PSMA PET	42	Retrospective study	Intermediate and high risk	≥7	93.3	96.3	93.3	96.3
Shiiba et al. (21)	^11^C-MET PET	20	Prospective study	All	≥6	61.2	89.7	n.m	n.m
Suzuki et al. (22)	^18^F-FACBC PET	55	Prospective study	Intermediate and high risk	≥6	64.5	99.6	n.m	n.m
Wit et al. (23)	SLN technique	2,509 (21 studies included)	Meta-analysis	All	All	95.2	100	100	98
Manny et al. (24)	ICG technique	60	Prospective study	All	n.m	100	75	15	100
Nguyen et al. (25)	ICG technique	42	Prospective study	Intermediate and high risk	n.m	80–98	57	8	95

*CT, Computer tomography; MRI, Magnetic resonance imaging; MRImp, Mutliparametric MRI; DWI, Diffusion-weighted imaging; SPIO, Superparamagnetic iron oxide; n.m, Not mentioned; PET, Positron emission tomography; ^18^F, Fluor-18; ^11^C, Carbone-11; ^68^Ga, Gallium-68; FDG, Fluorodeoxyglucose positron emission tomography; FEC, Fluoroethylcholine; PSMA, Prostate specific membrane antigen; MET, Methionine; FACBC, Fluorocyclobutane-1-carboxylic acid; SLN, Sentinel lymph node; ICG, Indocyanine green*.

## Evidence Synthesis

### Morphological and Functional Imaging (CT and MRI)

CT and MRI, with cross sectional imaging, use morphological characteristics for the nodal staging, such as size and shape (oval or round) of the node. The criteria used as indicators of metastatic node are a diameter, in short axis, >1 cm for oval nodes and >0.8 cm for round nodes (26). With such criteria, a meta-analysis found that for detection of positive nodes, CT has a pooled sensitivity of 42% and a specificity of 82% and MRI a sensitivity of 39% and a specificity of 82% (10), thus confirming the limited value of morphological exams in LN staging. MRI, however, offers the possibility to evaluate other nodal characteristics, such as the “Apparent Diffusion Coefficient” (ADC) calculated via diffusion-weighted imaging (DWI), which pictures the Brownian movements of water's molecule. Moreover, another technique currently employed in MRI for pathologic node detection is the use of superparamagnetic iron oxide (SPIO) nanoparticles that can be used as contrast agents for improving LNM detection sensitivity.

In DWI sequences, MRI may be performed without any contrast agent and malignant tissue tends to have restricted water diffusion due to increased cellularity, enlarged nuclei, macromolecular proteins in the cytoplasm and extracellular disorganization. Malignant lesions appear hyperintense at high coefficient values (b-value of 800–1,000 s/mm2) and hypointense on the ADC maps (lower ADC value). Nonetheless, DWI-MRI can misclassify necrotic lymph nodes due to free diffusion of water, which was described in a study on diagnostic accuracy of MRI of lymph node metastasis in the neck (27). A recent prospective study using the combination of morphological criteria and DWI sequence on a 3T MRI showed a specificity of 94% and a sensitivity of 41% for the detection of LNM (11).

In SPIO MRI, nanoparticles are avidly taken up by macrophages. When a lymph node is metastatic, there is a paucity of macrophages and therefore a reduced uptake of SPIO. SPIO causes a loss of signal on T2 weighted image; as such, benign lymph nodes appear dark while metastatic nodes appear bright. After injection of SPIO nanoparticles, the lymphatic areas are analyzed on T2-weighted fast spin-echo or gradient-echo sequences. The initial studies with SPIO were performed with Ferumoxtran-10; however, this compound did not receive FDA approval. Ferumoxytol, on the other hand, is a new SPIO agent that has been tested for detection of malignant lymph node, which already had FDA and EMA approvals for iron replacement therapy in patients with chronic kidney failure. SPIO nanoparticles increase the sensitivity compared to MRI alone from 45 to 100% with 95.7% specificity in the sentinel lymph node detection (28). They also demonstrated the ability to detect metastatic lymph nodes with < 7 mm diameter in short axis (29). The initial study using ferumoxtran-10 reported a sensitivity for detection of malignant lymph nodes with short axis diameter of 5–10 mm of 96.4% (compared to 28.5% with MRI alone) (12). However, Ferumoxytol is not approved for MR imaging and currently, SPIO MRI remains in research field with no clinical routine application.

The combination of DWI sequences and administration of SPIO represent promising techniques for the evaluation of LNM. The combination of DWI-MRI and SPIO contrast agent improves the sensitivity and the specificity and it decreases the time needed for interpreting images (30).

### PET Imaging for Lymph Node Staging

PET imaging offers a better spatial resolution than planar scintigraphy and single photon emission computed tomography (SPECT). Multiple radiotracers have been explored and are currently tested in the field of prostate cancer.

#### ^18^F-Fluoro-Desoxy-Glucose

^18^F-Fluorodeoxyglucose (^18^F-FDG), the most used in oncological investigations, that explores glucose consumption by malignant cells with increased glycolytic activity, has limited utility in PCa due to low metabolic activity of PCa cells particularly in the early phase of disease (31). It can be useful for the detection of metastatic lesions (32) or in poorly differentiated PCa (33). A study analyzing intermediate and high risk PCa, reported that ^18^F-FDG PET had a sensitivity of 80% and a positive predictive value (PPV) of 87% for detection of primary lesion in case of Gleason score ≥7 (34). In the Beauregard et al. study on pre-operative staging with FDG PET for high grade PCa, 41 patients had ePLND, 11 of them had LN metastasis at histological analysis but FDG PET detected only 3 of those (27%) with LNM pathology-proven, if non-surgical LN FDG were included and suspicion confirmed by response to ADT, the sensitivity were evaluated to 47%. Moreover, FDG PET found 7 patients with suspected LNM but only 3 were confirmed histologically (13). In the Chang et al. study on the role of FDG PET to detect LNM in case of PSA relapse after treatment for localized PCa, they find a sensitivity, specificity, PPV and NPV of 75, 100, 100, and 67.7%, respectively (14). The literature is limited on the diagnostic accuracy of FDG PET in LN staging for PCa, but it still probably have a place in the staging for advanced PCa with high Gleason score or undifferentiated PCa. A further disadvantage of ^18^F-FDG is the urinary excretion of this tracer that can potentially decrease the sensitivity for the detection of pelvic lesions. Therefore, ^18^F-FDG PET plays a particularly limited role in the detection of pelvic lymph node metastases.

#### Choline Derivate Radiotracers

Other radiotracers have been studied for PCa imaging at initial staging and BCR. The most studied are choline derivatives radiolabeled with Carbone-11 (^11^C) or Fluor-18 (^18^F). Choline is naturally incorporated into tumor cells after phosphorylation by choline kinase that is upregulated in malignant PCa cells (35). Choline derivatives show minimal urinary excretion and therefore less activity in the bladder compared to ^18^F-FDG.

In a meta-analysis ^11^C-Choline PET presented a performance for detecting lymph node metastases with a pooled sensitivity of 58% and a pooled specificity of 94%. ^18^F-Choline, on the other hand, had a lower sensitivity (40 vs. 58% for ^11^C-Choline, respectively) but improved specificity (96 vs. 94% for ^11^C-Choline) (15). Tilki et al. performed a study on detection rate for ^18^F-Fluoroethylcholine with a lesion-based analysis in 1,149 lymph nodes. The result was a sensitivity of 39.7% and a specificity of 95.8% with a NPV of 83% and a PPV of 75.7%, respectively (16). Globally, ^11^C- or ^18^F- radiolabeled choline derivatives present similar performances for detection of LN invasion. However, detection rates increase with increasing PSA serum levels and in the Mitchell et al. study they found that the optimal PSA serum level for lesion detection in case of BCR was a value ≥2 ng/ml (36). Currently, the EAU recommend PET imaging with choline derivatives for BCR after radical prostatectomy with PSA serum level ≥1 ng/ml (37).

#### Acetate PET Imaging

Acetate PET imaging explores the fatty acid metabolic pathway which is overexpressed in PCa (38). Acetate is a naturally present fatty acid precursor and is converted to acetyl-CoA that is incorporated into cholesterol and fatty acids. Most studies have been performed using ^11^C-Acetate PET imaging. The major elimination route is through the respiratory system with a lack of significant renal activity (39). Therefore, imaging can be performed without interference of urinary activity. Nonetheless, as ^11^C presents a half time of 20 min, its use is limited to centers equipped with a cyclotron.

Schumacher et al. analyzed the performance of ^11^C-Acetate PET/CT: Sensitivity, specificity, PPV and NPV were calculated at 90, 67, 75, and 86%, respectively. The nodal-region-based sensitivity was 62% with a specificity of 89% and a PPV of 62% with a NPV of 89% (17). Haseebuddin et al. evaluated ^11^C-Acetate PET/CT in 107 men with intermediate and high-risk PCa, reporting a performance for pathologic lymph node involvement with a sensitivity of 68% and a specificity of 78% with a PPV of 49% and a NPV of 89% (18). Compared to choline derivative PET imaging, studies found no significant difference in detection rate between ^11^C-Acetate and ^18^F-Choline PET/CT in case of BCR with low PSA level (40). To date there is no clear evidence that ^11^C-Acetate PET/CT performs better than choline derivatives in lymph node staging, and both tracers present increased detection with increasing PSA serum level.

#### Prostate Specific Membrane Antigen (PSMA) Imaging

PSMA is a transmembrane type II glycoprotein also called folate hydrolase I or glutamate carboxipeptidase II, which has a 19-amino-acid intracellular portion, a 24-amino-acid transmembrane portion and a 707-amino-acid extracellular portion (41). PSMA is expressed by prostate cells and overexpressed in PCa cells. The first PSMA imaging agent used was ProstaScintTM, an ^111^Indium-labeled monoclonal antibody. This PSMA target was an intracellular domain of PSMA, therefore targeting antigens only accessible in apoptotic or necrotic cells (not viable tissue) where the membrane was permeable with limited sensitivity and specificity (42, 43). Furthermore, the ProstascintTM showed slow kinetics requiring imaging 5–7 days after the injection.

Recently, novel small molecules targeting the extracellular domain of PSMA have been developed such as:

- N-{N-[(S)-1,3-dicarboxypropyl] carbamoyl}-4-(^18^F)fluoro benzyl-L-cysteine [(^18^F)DCFBC],
- ^68^Ga-N,N′-bis [2-hydroxy5-(carboxyethyl)benzyl] ethylenediamine-N,N′-diacetic acid (HBED-CC)
- 2-(3-(1-carboxy-5-[(6-[^18^F]fluoro-pyridine-3-carbonyl)-amino]-pentyl)-ureido)-pentanedioic acid ([^18^F]DCF PyL)
- EuK-Subkff-^68^Ga-DOTAGA (^68^Ga-PSMA I&T)

The most widely used and studied PSMA tracer is ^68^Ga-PSMA (HBED-CC), with multiple PET/CT studies analyzing the diagnostic value of PSMA ligand radiotracer in lymph node detection in case of BCR. Budaus et al. retrospectively evaluated the diagnostic value of PSMA PET/CT in preoperative high-risk PCa. The overall sensitivity, specificity, PPV, and NPV were 33.3, 100, 100, and 69.2%, respectively (19). In their study, the authors also reported that the detection rate varied with the median size of LNM. The false negative LN metastases had a median diameter of 4.3 mm (vs. 13.6 mm for the positive nodes) (19).

Zang et al. explored PSMA in intermediate and high-risk PCa: the pre-operative ^68^Ga-PSMA PET/CT lymph node diagnostic value showed a sensitivity of 93.3% with a specificity of, 96.3%, and a PPV of 93.3% with a NPV of 96.3%, on patient-based analysis. On LN-based analysis, sensitivity, specificity, PPV and NPV were 96.1, 99.6, 96.1, and 99.6%, respectively (20).

In PCa patients with BCR, Eiber et al. found detection rates of 96.8, 93.0, 72.7, and 57.9% for PSA values of ≥2, 1 to < 2, 0.5 to < 1, and 0.2 to < 0.5 ng/mL, respectively, and detection rates increased with a higher PSA velocity to 81.8, 82.4, 92.1, and 100% in < 1, 1 to < 2, 2 to < 5, and ≥5 ng/mL/year, respectively (44).

In a large retrospective study by Afshar-Oromieh et al. that included 319 PCa patients with BCR, a correlation of rate detection was found in multivariate analysis with PSA level and androgen deprivation therapy (ADT), probably linked with a PSMA expression by PCa cells upregulated by ADT. No correlation was found with Gleason score (compared to low Gleason score of 5–6) or PSA doubling time (45). Furthermore, in a multicenter prospective study on the impact of ^68^Ga-PSMA PET/CT in the management of PCa, Roach et al. found that PSMA ligand imaging detected additional nodal disease compared to conventional imaging in 39% patients at sites previously unknown and led to a change in planned management in 51% of patients (46).

Nonetheless, PSMA ligand uptake can be seen in other malignancies (glioblastoma, hepatocellular carcinoma, lung cancer, renal cell cancer, thyroid cancer, follicular lymphoma) and benign conditions (schwannomas, thyroid adenoma, hemangioma, sarcoidosis, inflammatory lymph node). This requires interpretation of data by a dedicated nuclear medicine team, capable of correctly interpreting the images of these PET examinations. Moreover, a major limitation of most PSMA studies is the lack of histological confirmations of positive lesions. As such, what investigators “see” as PCa metastases on imaging may not necessarily correspond to pathologic disease, albeit extremely high specificity rates were reported in studies in which histological confirmation was obtained (19).

### Amino Acid Imaging

Amino acid transport and metabolism is upregulated in malignant cells, including PCa cells. Various amino acid transporter systems or pathways can be targeted. The first radiolabeled amino acid studies in PCa were involving ^11^C-Methionine and ^11^C-hydroxy-Tryptophane. They showed excellent and promising results, although in few and small series, which served nonetheless as a proof of concept (21, 47, 48, 49, 50).

Other non-natural amino-acids have been synthesized and the most studied is the anti-1-amino-3-18F-fluorocyclobutane-1-carboxylic acid (^18^F-FACBC or ^18^F-Fluciclovine). Fluciclovine has minimal urinary excretion and minimal brain uptake which allow imaging of cerebrum, retroperitoneum and pelvis. The highest uptake is seen in pancreas, liver, bone marrow, salivary glands, lymphoid tissues and pituitary as found with other amino acid based radiotracers. In a multicenter phase IIb clinical trial, ^18^F-Fluciclovine PET/CT was compared to conventional imaging with CT and bone scan. The overall accuracies were similar for ^18^F-Fluciclovine PET/CT and conventional imaging (85.5 vs. 87.3%). However, ^18^F-fluciclovine PET/CT was positive in 5–9 mm lymph nodes that were not detected by conventional imaging (22). Currently, ^18^F-Fluciclovine seems to present promising results in the characterization of primary disease as the uptake is significantly higher in PCa compared to normal prostate tissue (51).

### The Sentinel Lymph Node Techniques in Prostate Cancer

The sentinel lymph node dissection techniques rely on the principle that by detecting the primary nodal landing sites where tumor cells would spread by following the lymphatic pathway and by analyzing if there is a tumor invasion in these site a limited and less invasive lymph node dissection could be performed, thereby reducing the morbidity caused by an extensive lymph node dissection. In uro-oncology, the sentinel lymph node (SLN) technique was first described for the penile cancer in 1977 (52). In PCa the first study was reported in 1999 by Wawroschek et al. (53).

Albeit current international guidelines do not recommend performing SLN techniques for PCa (3), this is a field of active research, especially with the introduction of indocyanine green and fluorescence guided surgery. The two most widely used SLN techniques are the radio-isotope injection of ^99m^Tc-nanocolloid and fluorescence imaging with the indocyanine green.

#### Radio-Isotope SLN Technique

In the ^99m^Tc-nanocolloid technique, the radiotracer is injected transrectally in the prostate. Lymphoscintigraphy with static planar acquisitions are performed 15 min and 2 h after the injection followed by SPECT/CT imaging. Thereafter, during surgery, the SLN is detected using gamma probes. The technique has been developed over the last 15 years, and is routinely used in breast cancer and melanoma surgery (54, 55). The technique is feasible in PCa patients and Holl et al. demonstrated that SLN could be detected in up to 98.2% of patients with clinically localized PCa disease. They conducted a large study in 2020 clinically localized PCa patients. Preoperative planar lymphoscintigraphy detected drainage patterns in 97.6% of patients while intraoperative SLN detection using a handheld probe was successful in 98% of cases. They reported a false negative rate of 5.9% with an increase up to 14% in patients with Gleason > 8 (56).

In a recent systematic review of SLN in PCa, the overall median pre-operative and intraoperative detection rates were 97.8 and 96.1%, respectively, with an overall median false negative rate of 7.1% (57).

Wawroschek et al. demonstrated the validity of the technique with a 97% sensitivity to detect the location of lymphatic spread in node positive patients and improved detection of micrometastases (53, 58, 59). Two meta-analyses suggested a sensitivity around 95% to detect LNM (23, 60). The most common areas of LN metastases detected with SLN technique is the external and internal iliac regions, followed by the obturator, common iliac, and presacral regions. Frequently, the SLN technique detected bilateral LN (56, 61, 62).

Nonetheless, the SLN technique presents major limitations, hampering its introduction in routine surgical practice. First of all, there is a lack of standardized protocol (number and sites of injections) and difficulties for the intra-operatory detection with gamma probe (distance between the lymph node and probe in open surgery, large gamma cameras not adequate in robotic-assisted surgery). Second, the false negative rate may be due to an obliteration or obstruction of lymphovascular vessels and nodes by metastatic cells. Other causes of false negative results are the modifications of lymphatic drainage due to previous treatments (surgery, TRUS, radiotherapy) or inflammation and infection conditions. In such situations, the SLN technique should be analyzed with caution.

#### Fluorescence Imaging

For the indocyanine green (ICG) technique, ICG is also injected in the prostate via a transrectal route and the SLN is detected preoperatively with polarized light. It can be injected before the surgery or intra-operatory. The ICG present the advantage to be non-radioactive. It has been used for more than 50 years for hepatic clearance and cardiovascular function evaluation as well as in ophthalmic angiography, with few reported allergic reactions to its iodine content. It undergoes rapid hepatic metabolism and excretion in bile. In uro-oncology, the first study with ICG was reported in 2011 by Inoue et al. in 14 patients with open radical prostatectomy (63). Since then, different studies have been published with the ICG technique and the median intraoperative fluorescence detection rates ranged between 76 and 97% with a sensitivity of 100% for metastatic lymph node detection (24, 25, 64, 65). Chennamsetty et al. performed an ICG lymph node mapping during robotic assisted radical prostatectomy in 20 patients with high or intermediate risk PCa. They found a sensitivity, specificity, PPV and NPV of 62, 50, 8, and 95%, respectively (66).

Van der Poel et al. were the first, in 2011, to demonstrate the feasibility of an hybrid fluorescent-radiotracer technique in 11 patients undergoing robotic radical prostatectomy. They used a colloid to which the ICG was bound additionally to form an “hybrid tracer” (67). Jeschke et al. published a study of 26 patients undergoing laparoscopic lymph node dissection guided by hybrid fluorescent-radiotracer. In their study, 18 h before surgery the patient received the conventional radio-isotope intraprostatic injection and ICG was then injected intra-operatively (68). Both studies concluded that the combination of tracer improves the detection rate and the ICG added the advantage of visualizing the lymphatic vessels in real time.

The ICG technique present the same accuracy and limitations as the conventional radio-isotope technique and the study by Chennamsetty et al. found a poor diagnostic accuracy of SLN technique in high and intermediate risk PCa probably in relation with the metastatic obliteration of lymphovascular vessels and nodes (66). Due to the complex drainage pattern of the prostate, and the low sensitivity of the technique for the detection of nodal metastases, fluorescence SLN detection is not enough reliable, at present, to be considered as an alternative to an accurate PLND in higher risk patients.

### Innovative Techniques

Winter et al. reported on the first human study using superparamagnetic iron oxide (SPIO) nanoparticles as magnetic SLN tracer in 2014 (28). They published a detection rate of 90% with a 100% sensitivity for metastatic nodes. The SPIO seems to improve the sensitivity of MRI from 45 to 100% with a specificity of 97%. In a feasibility study with PSMA-labeled radiotracer (^111^In-PSMA-I&T) for SLN, Maurer et al. detected in five patients 2–4 mm LN lesions and additional nodes that ^68^Ga-PSMA PET did not detect (69). As such, this PSMA-labeled radiotracer could facilitate lymphadenectomy in patients with very high-risk and oligometastatic PCa, in which metastatic LNs have initially been detected by PSMA imaging.

Finally, in *in vitro* model and in a murine model, the feasibility of SPIO nanoparticle-bound PSMA ligands has been shown, with an increased uptake of SPIO by PCa cells lines that expressed PSMA (70, 71). This represents a potential tracer for the evaluation of lymph node and metastatic extension in PCa with PET/MR device. Further clinical investigations are required.

## Conclusions

Optimal lymph node staging in prostate cancer remains an important goal in the management of patient with PCa at pre-operative evaluation and for BCR investigation. Molecular imaging, particularly PSMA ligand PET imaging, presents interesting diagnostic accuracy in LN diagnosis even in subcentimetric LN particularly in low PSA value BCR. Nonetheless, the clinical significance of positive PSMA PET/CT findings, and its impact on oncologic outcomes, remains to be defined. Other PET tracers have been developed and continued to be studied. DWI-MRI presents good results in LN involvement evaluation and the use of contrast agent such SPIO may improve the detection rate. The SLN technique is limited to experimental protocols and for intermediate or high-risk PCa. Well-designed, prospective trials are needed to determine the place of novel imaging techniques and their clinical impact in management and decision making for PCa patients.

## Author Contributions

All authors listed have made a substantial, direct and intellectual contribution to the work, and approved it for publication.

### Conflict of Interest Statement

The authors declare that the research was conducted in the absence of any commercial or financial relationships that could be construed as a potential conflict of interest.
